# Novel Nuclear Protein Complexes of Dystrophin 71 Isoforms in Rat Cultured Hippocampal GABAergic and Glutamatergic Neurons

**DOI:** 10.1371/journal.pone.0137328

**Published:** 2015-09-17

**Authors:** Rafael Rodríguez-Muñoz, María del Carmen Cárdenas-Aguayo, Víctor Alemán, Beatriz Osorio, Oscar Chávez-González, Alvaro Rendon, Dalila Martínez-Rojas, Marco Antonio Meraz-Ríos

**Affiliations:** 1 Departments of Physiology, Biophysics and Neurosciences, Center for Research and Advanced Studies of the National Polytechnic Institute (CINVESTAV-IPN), México D.F., México; 2 Molecular Biomedicine, Center for Research and Advanced Studies of the National Polytechnic Institute (CINVESTAV-IPN), México D.F., México; 3 Institut de la Vision, UMR Inserm, Laboratoire de Physiopathologie Cellulaire et Moléculaire de la Rétine, Université Pierre et Marie Curie, Paris, France; University of Louisville, UNITED STATES

## Abstract

The precise functional role of the dystrophin 71 in neurons is still elusive. Previously, we reported that dystrophin 71d and dystrophin 71f are present in nuclei from cultured neurons. In the present work, we performed a detailed analysis of the intranuclear distribution of dystrophin 71 isoforms (Dp71d and Dp71f), during the temporal course of 7-day postnatal rats hippocampal neurons culture for 1h, 2, 4, 10, 15 and 21 days *in vitro* (DIV). By immunofluorescence assays, we detected the highest level of nuclear expression of both dystrophin Dp71 isoforms at 10 DIV, during the temporal course of primary culture. Dp71d and Dp71f were detected mainly in bipolar GABAergic (≥60%) and multipolar Glutamatergic (≤40%) neurons, respectively. We also characterized the existence of two nuclear dystrophin-associated protein complexes (DAPC): dystrophin 71d or dystrophin 71f bound to β-dystroglycan, α1-, β-, α2-dystrobrevins, α-syntrophin, and syntrophin-associated protein nNOS (Dp71d-DAPC or Dp71f-DAPC, respectively), in the hippocampal neurons. Furthermore, both complexes were localized in interchromatin granule cluster structures (nuclear speckles) of neuronal nucleoskeleton preparations. The present study evinces that each Dp71’s complexes differ slightly in dystrobrevins composition. The results demonstrated that Dp71d-DAPC was mainly localized in bipolar GABAergic and Dp71f-DAPC in multipolar Glutamatergic hippocampal neurons. Taken together, our results show that dystrophin 71d, dystrophin 71f and DAP integrate protein complexes, and both complexes were associated to nuclear speckles structures.

## Introduction

Duchenne muscular dystrophy (DMD) is an inherited disorder, characterized by progressive muscle degeneration, due to the absence of dystrophin [1]. Dystrophin 71 (Dp71) is the most abundant C-terminal form of dystrophin in the central nervous system (CNS), and two Dp71 DMD gene products (Dp71d and Dp71f) are generated by splicing of exon 78 [2]. Dp71d preserves the last 13 amino acids encoded by exon 78, whereas Dp71f lacks exon 78, and instead contains 31 novel amino acids (founder sequence) in its C-terminal region [3, 4]. In plasma membrane of neurons, both Dp71 isoforms bind dystrophin-associated proteins (DAP), to assemble Dp71-associated protein complexes [5–7]. DAP are a multiprotein complex integrated by: dystroglycans (DG), dystrobrevins (DB), syntrophins (SYN) and sarcoglycans (SAR). DG are glycosylated proteins composed by α- and β-subunits [8]. α-DG is an extracellular protein and may function as a receptor that detects the presence of laminin protein. β-DG interacts with laminin an adhesion protein in the extracellular matrix, through α-DG, and with cytoskeletal actin in the cytoplasm, directly or through Dp71, providing a structural link between the inside and the outside of the cell [9, 10]. DB and SYN are associated to the C-terminal region of Dp71 [11, 12]. SAR are four transmembranal proteins that have been widely studied in retina [13]. It has been shown that Dp71s are necessary for the stability of DAP complex in the plasma membrane of brain and retinal neurons, as well as glial cells [14, 15].

It is known that C-terminal mutations in DMD gene, that alter the expression of Dp71, are linked to a severe incidence of cognitive impairment of patients with DMD [16, 17]. Dp71 is mainly expressed in olfactory bulb, cerebral cortex, dentate gyrus and in CA regions of the hippocampus, which are brain areas involved in memory functions [18]. The presence of Dp71 was recently demonstrated at the glutamatergic synapse, in hippocampal neurons [17]. In spite of these observations, the precise functional role of the Dp71 in neuronal cells remains elusive.

The purpose of this study was to investigate a more precise intranuclear localization of Dp71 isoforms during the temporal course of the hippocampal neurons culture, and the possible formation of Dp71-DAP complexes (Dp71s-DAPC) in these primary cultures. To this end, we cultured hippocampal neurons for 0 (1h), 2, 4, 10, 15 and 21 days *in vitro* (DIV) from 7-day postnatal rats. We also purified nuclei and obtained subnuclear fractions (nucleoskeleton and chromatin) from cultured hippocampal neurons at 21 DIV. We used this time of culture, to study the subnuclear distribution of Dp71 isoforms, since at this time of *in vitro* culture, the hippocampal neurons are functionally mature [19]. In the present work, we found that the nuclear expression of Dp71 isoforms varied along the time of culture, but the highest level of nuclear expression of both Dp71 proteins was at 10 DIV. Additionally, we demonstrated that Dp71d and Dp71f were mainly localized at bipolar GABAergic (60%) and multipolar Glutamatergic neurons (40%), respectively. Dp71 isoforms were colocalized with α-DG, β-DG, α1-, α2-DB, β-DB, α-SYN, and nNOS, in nuclei, as tiny spots (nuclear speckles), from cultured hippocampal neurons at 21 DIV. Both Dp71 isoforms were bound to β-DG, α1-DB, β-DB, α2-DB, α-SYN, and nNOS, forming the respective complexes, in nucleoskeleton fraction of these hippocampal neurons, but each Dp71-DAPC differs slightly in DB composition. Dp71d-DAPC and Dp71f-DAPC were associated to the nucleoskeleton preparations of bipolar GABAergic or multipolar Glutamatergic hippocampal neurons, respectively. The interchromatin granule cluster structures or nuclear speckles are nuclear domains enriched in pre-mRNA splicing factors as well as factors involved in mRNA production by RNA polymerase II, supporting their intimately relationship to gene expression [20, 21].

Collectively, our data support the view that in the nucleus of hippocampal neurons: (i) Dp71d and Dp71f reached their highest level of nuclear expression at 10 DIV, during the temporal course of the neuronal culture; (ii) Dp71d or Dp71f together with DAP, integrate each a protein complex in the nuclei; (iii) Dp71-DAP complexes were differentially localized in bipolar GABAergic or multipolar Glutamatergic hippocampal neurons; (iv) the Dp71-DAP complexes are localized in nuclear speckles associated to the nuclear skeleton; and (v) the nuclear localization or composition of these complexes probably determine specific characteristics of each neuronal phenotype. These nuclear protein complexes that appears to be structurally similar to those present in the plasma membrane, offer a new functional alternative to Dp71 isoforms, not only involved in the plasma membrane and cytoskeletal architecture but probably also, in a nuclear process that determine and regulate the peripheral function of these complexes.

## Materials and Methods

All procedures were in accordance with the current Mexican legislation, NOM-062-ZOO-1999 (SAGARPA), based on the Guide for the Care and Use of Laboratory Animals. The Center for Research and Advanced Studies of the National Polytechnic School (CINVESTAV-IPN) Institutional Animal Care and Use Committee (IACUC), Mexico City, approved our procedures for animal use (protocol # 091–02).

### Ethics Statement

All efforts were made to minimize animal suffering.

### Animals

All animals were maintained under constant room temperature (RT; 23°C), 12 h—12 h light-dark cycle, with food and water *ad libitum* (S1 ARRIVE Checklist). Female Postnatal day 7 Wistar rats (10–15 g of body weight), bred in our facilities, were sacrificed according to the Mexican official norm NOM-062-ZOO-1999 for the production, care, use and sacrifice of laboratory animals. Briefly, 5 female rat pups of 7 days postnatal, were anesthetized (at noon) via intraperitoneal Pentobarbital injection (30 to 40 mg/kg, i.p.) and after that were decapitated, and the brains were dissected to obtain the hippocampal neurons for cell culture. See more details in the cell culture section of this Material and Methods.

### Antibodies

The properties and dilution of the antibodies used during this study are shown in Table 1.

**Table 1:** Characteristics of antibodies used in this study.

Antibody	Specificity	Position of antigen or sequence	Dilution used in Wb	Nature	Reference or Resource
Dys-2	Dp71d (with exon 78)	a.a. 3,669–3,685 of dystrophin	1:20	Monoclonal	Novocastra
5F3	Dp71f (founder sequence)	a.a. 3,698–3,704 of dystrophin	1:50	Monoclonal	[22]
Mandra-1	Dp427, Dp71d and Dp71f	a.a. 3,200–3,684 of dystrophin	1:500	Monoclonal	Sigma-Aldrich
K7	Utrophins	a.a. 3,161–3,388 of utrophin	1:3,000	Polyclonal	[23]
0030	α-DG	VHRRPQGDRAPARFKAKF	1:3,000	Polyclonal	Mornet D.
JAF	β-DG	PPPYVPP	1:1,000	Polyclonal	[24, 25]
Pan-DB	α1-, β-, α2-DB	a.a. 249–403 of DB	1:3,000	Polyclonal	Mornet D.
D124	α1-, α2-DB	DEAYQVSLQG	ND	Polyclonal	Mornet D.
β-Brevin	β-DB	DTVVSHMSSGVPTPTKRLQYS	ND	Polyclonal	Mornet D.
C4	α-SYN	RQPSSPGPTPRNFSEA	1:3,000	Polyclonal	[26]
sc-5602	GAD67	a.a. 1–101 of GAD67	ND	Polyclonal	Santa Cruz Biotech
sc-7609	GLUR1	C-terminus of GluR1	ND	Polyclonal	Santa Cruz Biotech
nNOS	nNOS	a.a. 1,095–1,289 of nNOS	1:1,000	Monoclonal	BD Trans-Lab
sc-15378	Emerin	a.a. 3–254 of emerin	1:4,000	Polyclonal	Santa Cruz Biotech
sc-20681	Lamins A/C	a.a. 231–340 of lamin A	1:4,000	Polyclonal	Santa Cruz Biotech
AV40922	Matrin-3	SKSFQQSSLSRDSQGHGRDLSAAGIGLLAAATQSLSMPASLGRMNQGTAR	1:1,000	Polyclonal	Sigma-Aldrich
sc-34263	Histone H4	acetylated serine 1 and lysine 5, 8 and 12 of Histone H4	1:1,000	Polyclonal	Santa Cruz Biotech
sc-15386	hnRNP C1/C2	a.a. 86–190 of hnRNP C1/C2	1:1,000	Polyclonal	Santa Cruz Biotech
sc-10252	SC35	N-terminus of SC35	ND	Polyclonal	Santa Cruz Biotech
sc-11397	Calnexin	a.a. 1–70 of calnexin	1:1,000	Polyclonal	Santa Cruz Biotech
sc-52385	CD4	Human CD4	ND	Monoclonal	Santa Cruz Biotech
Actin	All isoforms of actin	Central domain of actin	1:1,000	Monoclonal	[27]

a.a. = amino acids

ND = Not determined

### Cell cultures

Hippocampal neurons were cultured on coverslips (coated with Poly-D-Lys) during 0 (1h), 2, 4, 10, 15 and 21 days *in vitro* (DIV), as previously described [28]. The anti-mitotic compound cytosine β-d-arabinoside (Sigma-Aldrich) was added to neuronal cultures at a final concentration of 4.0 μM to prevent glial proliferation [29].

### Cell treatments

For heat shock experiments, Cells cultured in Petri dishes containing coverslips were switched to pre-warmed medium (45°C) and incubated for 15 min in a water bath preheated at 45°C, prior to fixation for immunofluorescence microscopy. Control cells were also seeded in the same type of Petri dishes with cover slips, and their culture medium was changed to pre-warmed 37°C medium and incubated for the same time at 37°C prior fixation.

### Nuclei isolation

Isolation of nuclei was essentially carried out as previously reported [28].

### Subnuclear fractions

Briefly, isolated nuclei were stripped of outer and inner nuclear membranes, by treatment in 200 μl of CSK-1 buffer (10 mM PIPES pH 6.8, 100 mM NaCl, 0.3 M Sucrose, 1.5 mM MgCl_2_, 1.0 mM EGTA and 0.5% [v/v] Triton X-100), followed by incubation on ice for 5 min. Membrane-depleted nuclei were collected by centrifugation at 5,000 × g for 5 min at 4°C, the supernatant contained the nuclear membranes fraction. Chromatin and soluble proteins were removed by resuspension of membrane-depleted nuclei in 200 μl of CSK-2 buffer (10 mM PIPES pH 6.8, 50 mM NaCl, 0.3 M Sucrose, 1.5 mM MgCl_2_, 1.0 mM EGTA and 15 U/ml RNase-OUT), containing 500 U/ml RNase-free DNase I and incubated 1 h at 30°C. Sample was centrifuged at 10,000 × g for 10 min at 4°C, the pellet contained the nucleoskeleton. The nucleoskeleton was resuspended in 200 μl of CSK-2 buffer containing 650 mM (NH_4_)_2_SO_4_ and incubated 5 min at 4°C, for removal of residual chromatin and soluble proteins. Sample was centrifuged as above described, and the pellet was resuspended in 200 μl of RIPA buffer (50 mM Tris-HCl pH 8.0, 150 mM NaCl, 2.5 mM EDTA pH 8.0, 1.0% [v/v] Triton X-100, 1% [w/v] Sodium deoxycholate and 0.1% [w/v] SDS). Nuclear fractions were boiled in the Laemmli buffer for 3 min, separated on a 12% (v/v) SDS-PAGE and immunodetected by Western blot (as described below). All the experiments were performed in presence of protein tyrosine phosphatases inhibitor (sodium orthovanadate [Na_3_VO_4_], Sigma-Aldrich), and protease inhibitor cocktail Complete (Roche Applied Science, Mannheim, Germany).

### Western blot analysis

Samples of total protein extracts and subnuclear protein fractions (100 μg) from cultured hippocampal neurons were resolved on 12% SDS polyacrylamide gel electrophoresis and electro-transferred to nitrocellulose membranes. Membranes were incubated for 1h with blocking buffer (3% [w/v] casein in 10 mM Tris-HCl pH 8.0, 150 mM NaCl, 0.05% [v/v] Tween-20), and then incubated overnight at 4°C with the appropriate Ab (Table 1). Immunoblots were developed using the western blot Luminol reagent (Santa Cruz Biotechnology). Membranes were stripped and reprobed with other Ab. Negative controls were incubated only with horseradish peroxidase-labeled secondary Ab without primary Ab.

### Immunoprecipitation

Immunoprecipitation of Dp71d (20 μl of Dys-2 Ab) and Dp71f (20 μl of 5F3 Ab) or both dystrophin isoforms (20 μl of Mandra-1 Ab) was performed with the nucleoskeleton fraction from cultured hippocampal neurons. Briefly, nucleoskeleton fraction (1 mg) was precleared with 20 μl of recombinant protein G-agarose beads overnight at 4°C. The beads were removed by centrifugation at 16,000 rpm for 5 min, and precleared extract was incubated overnight at 4°C with the immunoprecipitating Ab, previously bound to recombinant protein G-Agarose (Invitrogen, Carlsbad, CA, USA). As negative control, parallel incubation with a non-related Ab (monoclonal anti-CD4 Ab, as nonspecific immunoprecipitation) was performed. The immune complexes were collected by centrifugation at 16,000 g for 5 min, and washed three times for 10 min with 1 ml of RIPA buffer and then eluted by boiling in Laemmli buffer. Immunoprecipitated proteins were then analyzed by western blot as described above.

### Immunofluorescence and confocal microscopy assays

Cells were plated on coverslips and washed 2x in 1X GBS (5.4 mM KCl, 138 mM NaCl, 22 mM D-glucose, 2 mM Na-KPO_4_, pH 7.2) and fixed by addition of 4% (v/v) paraformaldehyde (Electron Microscopy Sciences), for 20 min at room temperature (RT), followed by 3x washes with GBS 1X. For immunocytochemistry fixed cells were permeabilized and washed three times in 1X PBS, pH 7.4 containing 0.2% (v/v) Triton x-100, 1% (v/v) (termed ICC Diluent), and incubated in blocking solution (1% Bovine serum albumin (v/v) in 1X PBS-0.2% Triton X-100) for 40 min at RT. Primary antibodies at the appropriate dilutions in ICC Diluent were incubated at 4°C ON, washed 3 x 10 min with ICC Diluent at RT, and incubated with Alexa-Fluor 488 or 594 fluorescently-labeled secondary antibodies diluted to 1:500 in ICC Diluent for 1h at RT in the dark. Coverslips were washed 3 x 10 min in ICC Diluent at RT and mounted on glass slides with Fluorogel (Electron Microscopy Sciences). Mounted coverslips were examined using a Leica laser scanning model SP8 confocal microscope (TCP-SP8, Leica, Heidelberg, Germany) and analyzed with LAS AF Image software, Ver. 4. For the colocalization analyze using the LAS AF software we selected the nuclear area or the cytoskeletal area and obtained the colocalization rate for that specific area of the cell. We measured 10 cells per each immunostaining condition, and calculate the average ± the SEM. Data was plotted using Graph Pad Prism 5.

### Nucleoskeleton Preparations

Nucleoskeleton preparations were obtained as previously reported [30], with some modifications. Briefly, cultured hippocampal neurons grown on coverslips at 21 DIV, were washed twice with ice cold PBS followed by incubation with CSK buffer (10 mM Pipes pH 6.8, 100 mM NaCl, 30 mM sucrose, 3 mM MgCl_2_, 1 mM EGTA, 0.5% (v/v) Triton X-100), to remove cytosolic soluble proteins. After 10 min at 4°C, the chromatin was eliminated by incubating with DNase I digestion buffer (CSK buffer containing 500 U/ml of RNase free DNase I) for 2h at 37°C. Next, to remove the remaining DNA and soluble nuclear proteins, the preparations were incubated with extraction buffer (CSK buffer containing 650 mM [NH_4_]_2_SO_4_) for 20 min at 4°C. Samples were washed with the CSK buffer and then, fixed with CSK and 4% (w/v) paraformaldehyde for 40 min in CSK. No staining of nucleoskeleton with DAPI was observed, demonstrating the effective removal of DNA by this procedure. Fixed nucleoskeleton was blocked for 20 min with 1.0% (w/v) IgG-free albumin (1331-A, Research Organics, Cleveland, OH, USA) in PBS, and incubated overnight at 4°C with primary Ab, diluted in PBS (see Table 1). Following incubation with the primary Ab, the samples were washed three times with PBS. Secondary Ab Alexa Fluor 488 donkey anti-mouse, Alexa Fluor 594 donkey anti-rabbit and Alexa Fluor 647 donkey anti-goat or Alexa Fluor 594 donkey anti-goat (Invitrogen, Carlsbad, CA, USA) were used; Secondary Abs were diluted 1:500 in PBS. Immunofluorescence was evaluated using a confocal inverted microscope (TCP-SP8, Leica, Heidelberg, Germany) and analyzed with the LAS AF Confocal software of Leica version 4.0. Immunofluorescence experiments were performed at least three times from three different experiments. Nonspecific labeling was assessed by exclusion of the primary Ab.

### Data analysis

Statistical analyze was performed using the Graph Pad Prism software, version 5.0. Results are expressed as mean ± standard error of the mean (SEM). Differences between means from two groups were evaluated by the Student’s t-test. One way analysis of variance (ANOVA) was used for multiple comparisons among groups. Bonferroni post hoc test was performed. ٭P˂0.05 and ٭٭P˂0.01 were considered to be statistically significant.

## Results

### Nuclear expression of Dp71 isoforms during the temporal course of cultured hippocampal neurons

**Fig 1:**
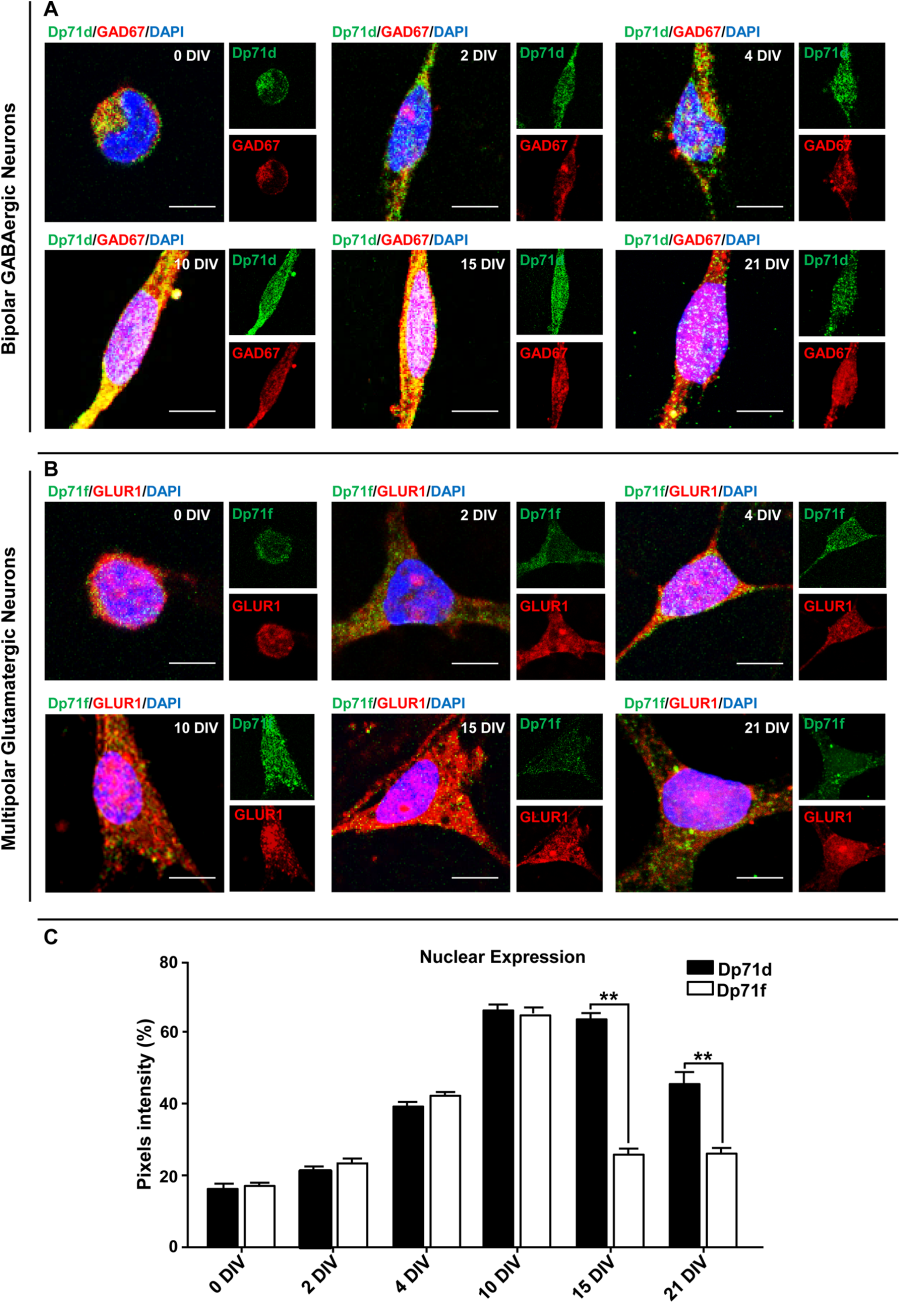
Nuclear localization of Dp71s during neuronal differentiation process. The temporal course of the nuclear expression of both Dp71s was performed, in primary cultures of hippocampal neurons. Hippocampal neurons were cultured for 0 (1h), 2, 4, 10, 15, and 21 days *in vitro* (DIV), and were double stained for Dp71d (Dys-2 Ab, green color, panel A) or Dp71f (5F3 Ab, green color, panel B) and GAD67 or GLUR1 Ab, respectively (red color, panel A and B). Both Dp71s were expressed in nuclei of neurons cultured for 0, 2, 4, 10, 15, and 21 DIV. Quantification of fluorescence intensity in the nucleus of each Dp71 is shown in panel C. The highest level of nuclear expression of both Dp71 proteins were at 10 DIV. Values are means ± SEM, ٭٭p˂0.01. Scale bar = 10 μm.

To assess if the subcellular distribution of Dp71 isoforms (Dp71s) change during the neuronal differentiation processes, we studied the temporal course of cultured hippocampal neurons, and evaluate the expression of both Dp71s in neuronal nuclei. By immunofluorescence assays with specific antibodies (Abs; see Table 1), we found that Dp71s were expressed in nuclei from neurons cultured for 0 (1h), 2, 4, 10, 15, and 21 DIV, but its level of expression varied along the time of culture (Fig 1). We detected a weak expression of nuclear Dp71s in fresh isolated primary hippocampal neurons seeded for only 1h (0 DIV; Fig 1C). After this time, we observed that the nuclear expression of both Dp71s increased over time of culture. It was found that the highest level of nuclear expression of both Dp71 proteins was reached at 10 DIV (Fig 1C). This level of expression of nuclear Dp71d was maintained through the 15 DIV, but at 21 DIV, a decrease of this isoform was observed; while the level of nuclear expression of Dp71f drops at 15 and 21 DIV, to a similar level reached at 2 DIV (Fig 1C).

**Fig 2:**
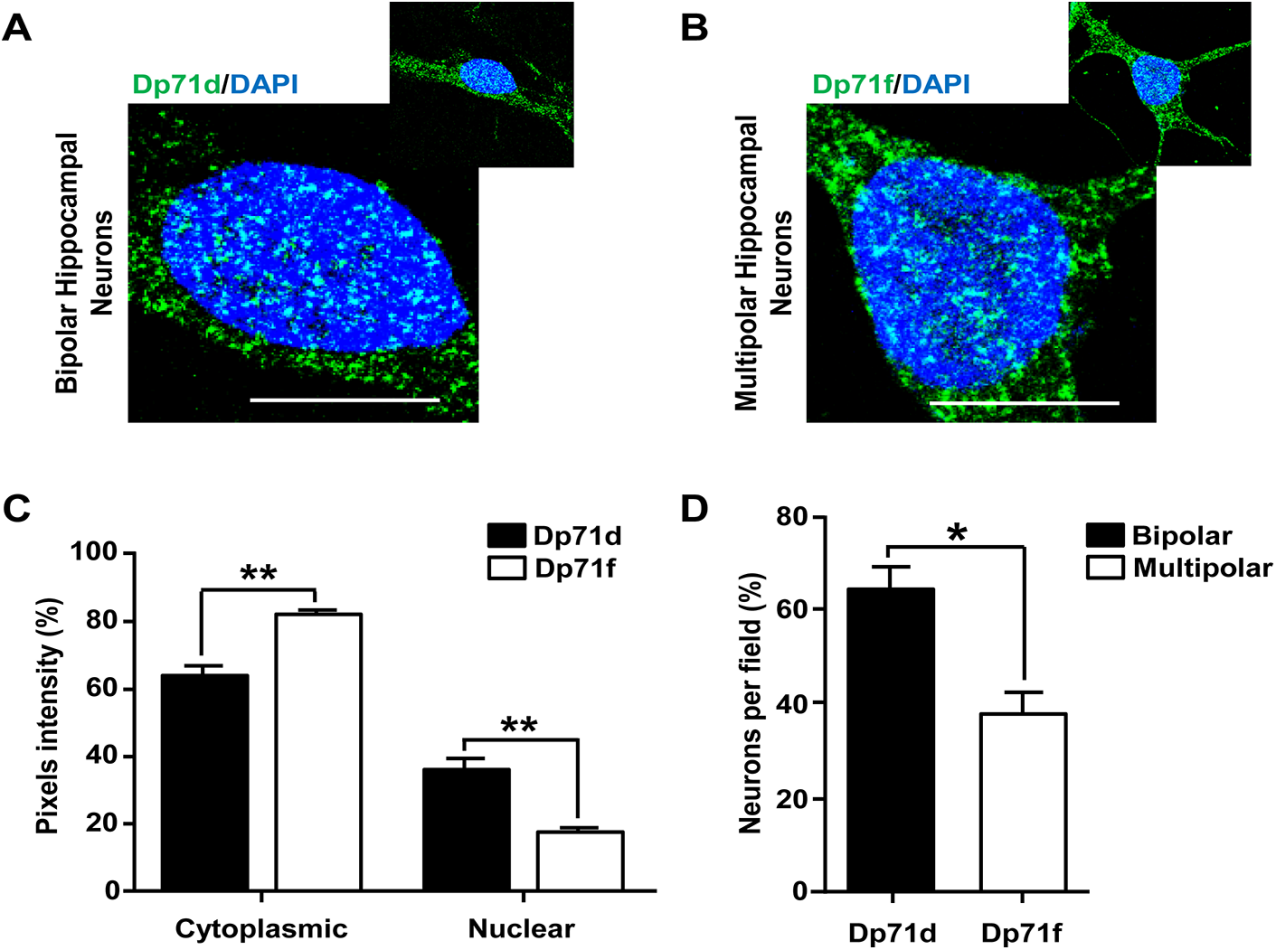
Localization of Dp71s in the nucleus of bipolar and multipolar hippocampal neurons. Cultured hippocampal neurons at 21 DIV were used for immunostaining assays. Subsequently, were fixed and immunoreacted with Dys-2 (Dp71d) or 5F3 (Dp71f) Ab and DAPI, respectively. Dp71d colocalized with DAPI into the nucleus of bipolar hippocampal neurons (green color, panel A). Whereas, Dp71f colocalized with DAPI into the nucleus of the multipolar hippocampal neurons (green color, panel B). Quantification of fluorescence intensity between cytoplasm and nucleus of each Dp71 is shown in panel C. The localization of each Dp71 at bipolar or multipolar neurons is shown in the panel D. Values are means ± SEM, ٭p˂0.05, ٭٭p˂0.01. Scale bar = 10 μm.

To analyze the nuclear distribution of Dp71s in primary cultures of hippocampal neurons, we identified the GABAergic or Glutamatergic phenotype of these cultured neurons by double immunostaining of Dp71s, with anti-glutamic acid decarboxylase 67 (GAD67, a marker of GABA neurons) or anti-glutamatergic receptor 1 (GLUR1, a marker of glutamate neurons) Abs, respectively (Fig 1A and 1B), and the Abs for the Dp71s (see methods). Dp71d was mainly expressed in bipolar GABAergic neurons (≥60%), whereas Dp71f was found in multipolar glutamatergic neurons (≤40%; Fig 2D). Additionally, both Dp71s were localized into the nucleus, as tiny spots (nuclear speckles-like granules) frequently present (Fig 1A and 1B). This nuclear speckles-like immunostaining was more evident in neurons cultured for 21 DIV; and the nuclear immunofluorescence of Dp71d was more intense than Dp71f (Fig 2). Collectively, these results show that Dp71d or Dp71f were localized in the nuclei of bipolar GABAergic or multipolar glutamatergic hippocampal neurons, respectively. Moreover, Dp71s were localized in nuclear speckle-like granules; and the Dp71d nuclear immunostaining was more intense than Dp71f in these primary cultures of hippocampal neurons at 21 DIV (Fig 2C). Furthermore we measured the colocalization of Dp71s with DAPI in the nucleus of primary hippocampal neuronal cells, to verify its nuclear localization (S1 Fig).

These results suggest that the presence of Dp71s in the nucleus of hippocampal neurons might be associated to the process of differentiation and the acquisition of morphological and physiological characteristics of the GABAergic or Glutamatergic cells. However, further investigation is required to explain this phenomenon.

### Subnuclear distribution of Dp71s and DAP in hippocampal neurons

**Fig 3:**
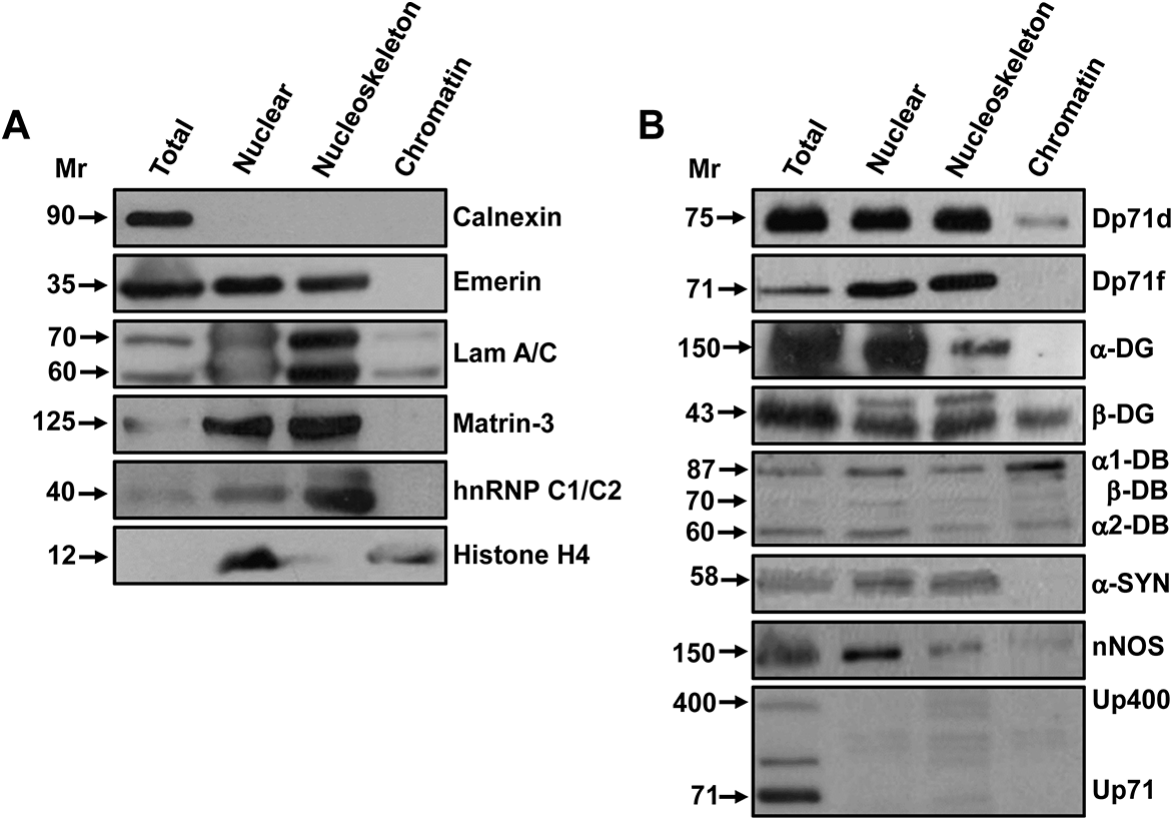
Expression of Dp71s and DAP in subnuclear fractions from hippocampal neurons. Protein samples from cultured hippocampal neurons at 21 DIV: total extract (Total), hippocampal nuclei (Nuclear), nucleoskeleton and chromatin, were obtained and analyzed by WB. In panel A, the specific protein markers of each subnuclear fraction are enriched in the corresponding sample: Calnexin for total extract, Emerin for inner nuclear membrane, Lamin A/C (Lam A/C), Matrin-3 and hnRNP C1/C2 for nucleoskeleton, and Histone H4 for chromatin. No contamination with Calnexin (endoplasmic reticulum marker) is observed in the nuclear fractions. In these conditions, using specific Abs (Table 1), the nuclear expression of Dp71s, and α-DG, β-DG, α1-, β-, α2-DB, α-SYN, nNOS and Utrophins (Up400 and Up71) is analyzed (panel B).

To gain more insight into the intranuclear distribution of Dp71s and DAP, we purified nuclei and obtained the nucleoskeleton and chromatin fractions from hippocampal neuron cultures at 21 DIV. The purity of each fraction was validated by Western blot (WB), and specific protein markers were detected: Calnexin for endoplasmic reticulum, Emerin for inner nuclear membrane, Lamins A/C (Lam A/C), Matrin-3 and hnRNP C1/C2 for nucleoskeleton, as well as Histone H4 for chromatin (Fig 3A). The subnuclear localization of each protein marker was observed in its respective fraction: nuclear, nucleoskeleton and chromatin, compared with the total cell extract (Total).

Subsequently, we analyzed by WB, using specific Abs (Table 1), the intranuclear expression of Dp71s, α-DG, β-DG, α1-DB, β-DB, α2-DB, α-SYN, neuronal nitric oxide synthase (nNOS), and utrophins (Up400 and Up71). As shown in Fig 3B, the total cell, nuclear and nucleoskeleton fractions presented a similar pattern of expression of Dp71d, Dp71f, α-DG, β-DG, α1-DB, β-DB, α2-DB, α-SYN, and nNOS. In the chromatin fraction was clearly found: β-DG and α1-DB; while Dp71d, β-DB, α2-DB; and nNOS were scarcely detected; and Dp71f, α-DG and α-SYN were totally absent. The dystrophin homologous proteins Up400 and Up71 were absent in the nuclear fractions analyzed.

To verify the nuclear expression of other dystrophin isoforms (Dp427) in hippocampal neurons cultured for 21 DIV, we compared total cell and nuclear extracts by WB, using Mandra-1 Ab (which detects Dp427 and both Dp71s). Dp71d and Dp71f were the only DMD gene products, expressed in nuclear extracts, as compared with the total extract (S2A Fig). Dp71d expression was more robust than Dp71f in the nuclear fraction (S2B Fig), and these data are consistent with the intensity of the immunofluorescence for each dystrophin shown in the Fig 2C.

These data showed that the Dp71s and DAP are co-expressed in nucleoskeleton fractions from primary hippocampal neurons cultured from 1h to 21 DIV.

### Dp71 isoforms and DAP integrate specific nuclear complexes in hippocampal neurons

**Fig 4:**
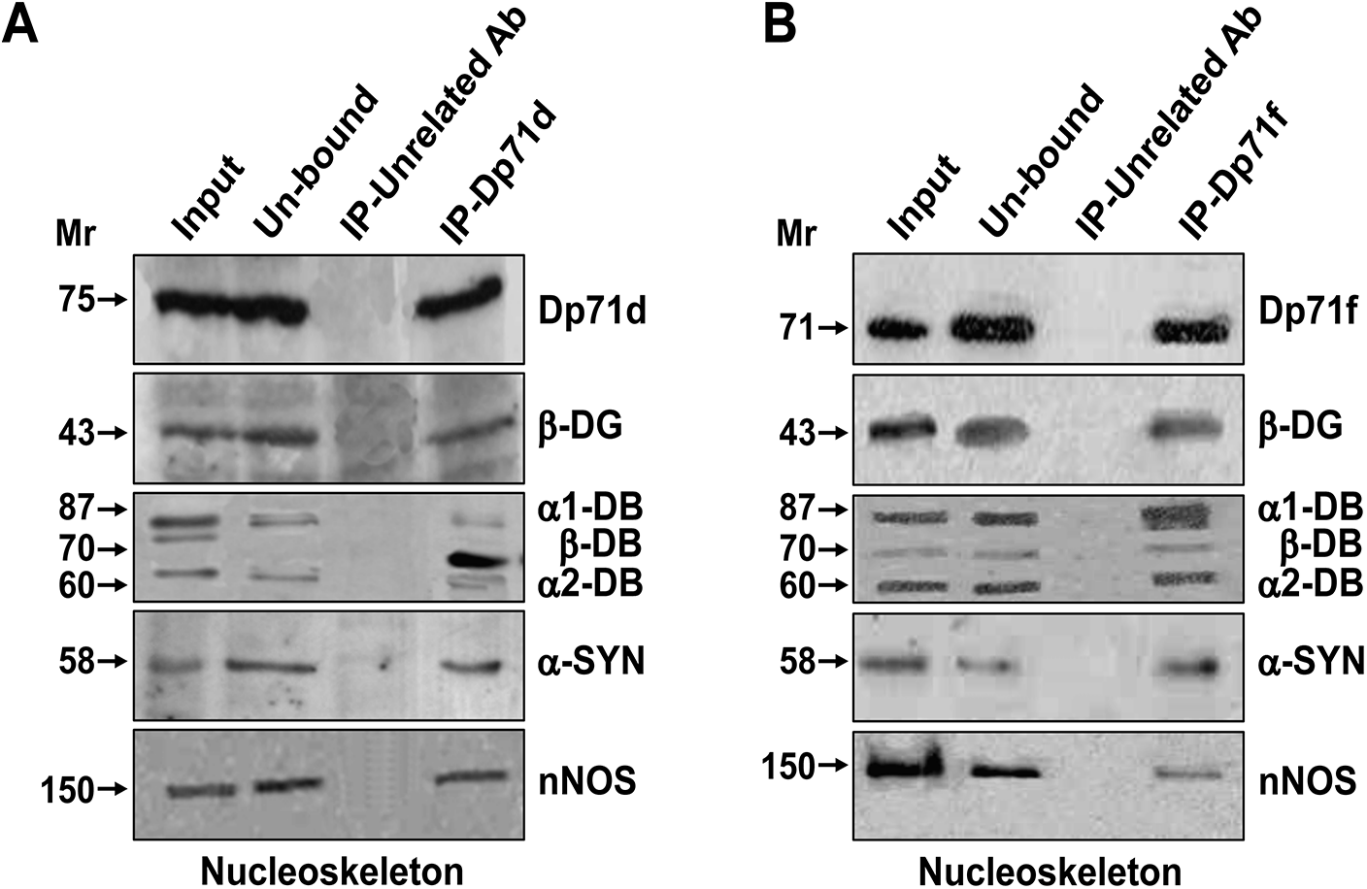
Dp71s-DAP complexes immunoprecipitated from nucleoskeleton fraction of hippocampal neurons. Nucleoskeleton fractions were immunoprecipitated (IP) with either Dp71d or Dp71f (Dys-2 or 5F3 Ab, respectively). Samples of the input nucleoskeleton fraction (Input), unbound and bound (IP-Dp71d or IP-Dp71f) proteins were analyzed by WB. The Dp71s, β-DG, α1-DB, β-DB, α2-DB, α-SYN and nNOS were detected as indicated in the right side. The immunoprecipitate from the nucleoskeleton fraction with Dp71d, contain a similar pattern of proteins than Dp71f. The only difference observed among Dp71s-DAP complexes was the pattern of associated dystrobrevins: Dp71d was preferentially bound to β-DB and Dp71f to α1-DB, α2-DB. As control for non-specific interactions, immunoprecipitation with CD4 (see Table 1), a non-related protein Ab was performed (IP-Unrelated Ab).

To further analyze, if Dp71d or Dp71f interact with DAP forming the respective complexes in the nucleoskeleton fraction; we carried out immunoprecipitation assays (IP). Each Dp71 precipitated was analyzed by WB, for the presence of Dp71d or Dp71f coupled to β-DG, α1-DB, β-DB, α2-DB, α-SYN and nNOS (Fig 4). We found that either Dp71s were associated to these proteins, forming the respective complexes (Dp71d-DAPC or Dp71f-DAPC). Additionally, we found that the pattern of associated dystrobrevins to each Dp71s-DAPC was different: Dp71d was preferentially bound to β-DB and Dp71f to α1-DB, α2-DB (Fig 4).

These results are consistent with the nuclear distribution of both Dp71s and DAP in the nucleoskeleton fraction (Fig 3B). Together, the immunofluorescence assays and IP data confirm that the Dp71s and DAP integrate two different complexes in the nucleoskeleton fraction, from bipolar GABAergic (expressing nuclear Dp71d) and multipolar Glutamatergic (expressing nuclear Dp71f) hippocampal neurons, respectively.

### Dp71s-DAP complexes localized in nuclear speckles are associated to nucleoskeleton preparations of hippocampal neurons

**Fig 5:**
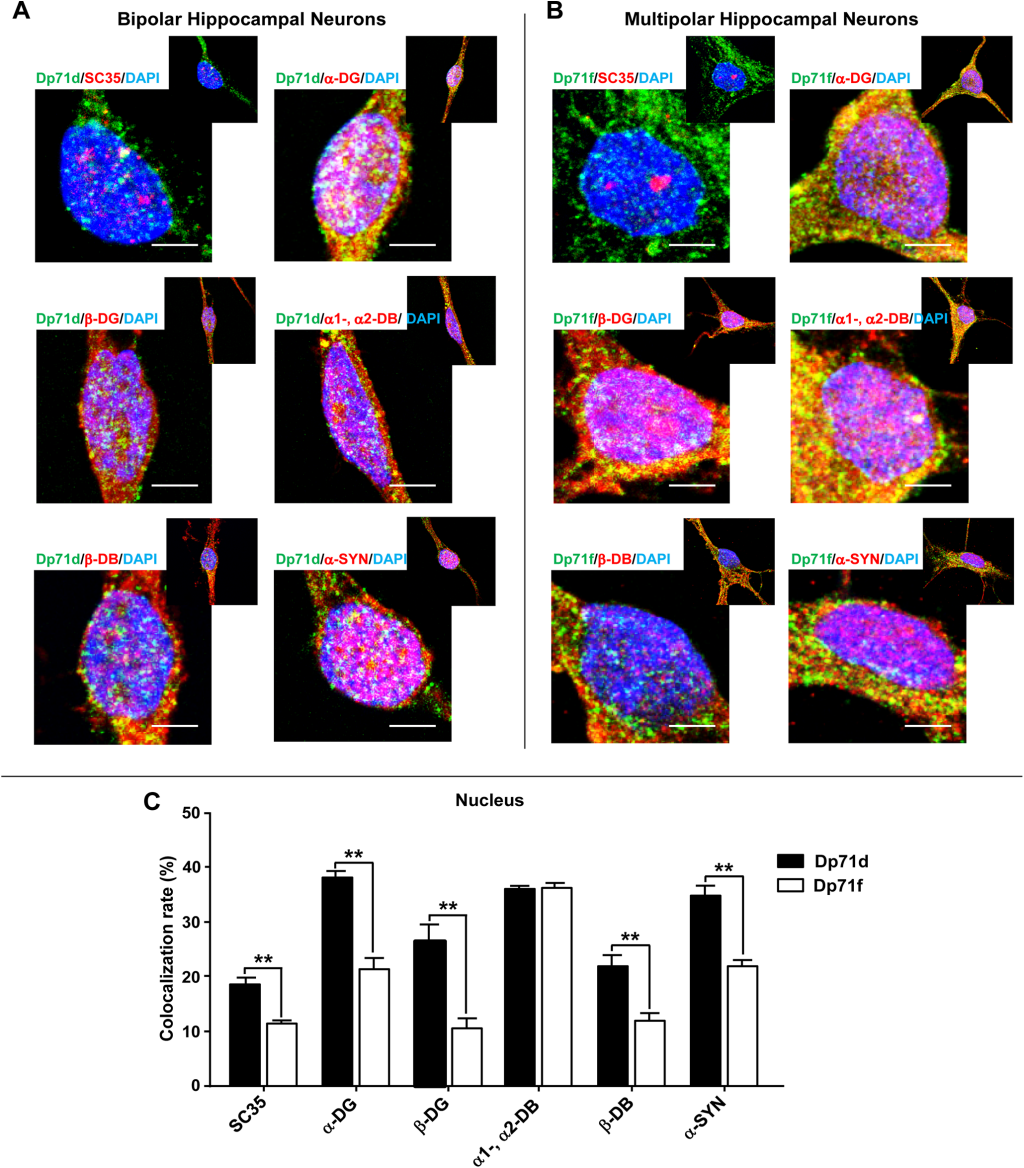
Colocalization of Dp71s with SC35 or DAP in bipolar and multipolar hippocampal neurons. Cultured hippocampal neurons at 21 DIV were used for immunostaining assays. Hippocampal neurons were double stained with either Dp71d (Dys-2 Ab, green color, panel A) or Dp71f (5F3 Ab, green color, panel B) and SC35, α-DG (K8), β-DG (JAF), α1-, α2-DB (D124), β-DB (β-Brevin), and α-SYN (C4) Abs, respectively (red color, panel A and B). DAPI was used to stain the nuclei, and the fucsia color indicates the colocalization of Dp71s and each DAP in nuclear speckles. The colocalization rate of each Dp71 with SC35 or DAP is shown in the panel C. Values are means ± SEM, ٭٭p˂0.01. Scale bar = 5 μm.

In eukaryotic cells, using immunofluorescence assays, the pre-messenger RNA splicing factors, small nuclear ribonucleoprotein and arginine/serine proteins have been localized in granule nuclear structures, termed SC35 domains, splicing-factor compartments or nuclear speckles [31–33]. Dp71d and Dp71f were observed, as tiny spots, into the nucleus, during the temporal course of the neuronal culture. This nuclear granule immunostaining was more evident in neurons cultured at 21 DIV. Accordingly, we analyzed the colocalization of Dp71s with DAP in these nuclear structures, by immunofluorescence and confocal microscopy assays. As shown in Fig 5, we found that the colocalization of Dp71d or Dp71f with SC35 (a nuclear speckle marker), α-DG, β-DG, α1-, α2-DB, β-DB and α-SYN was clearly observed in nuclear speckles of bipolar and multipolar neurons, respectively (Fig 5A and 5B), except for nNOS that was distributed in a more diffused way (data not shown).

**Fig 6:**
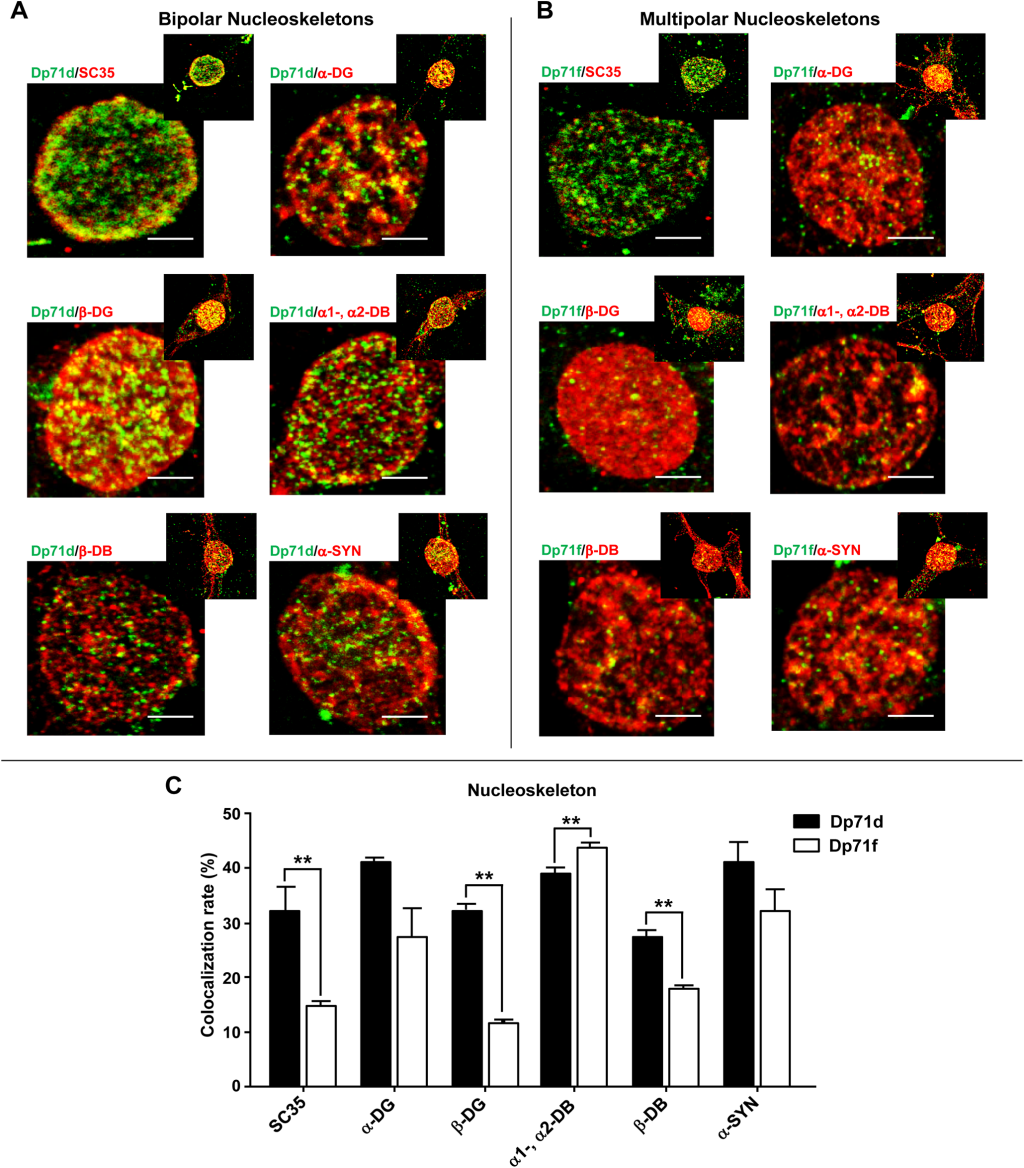
Colocalization of Dp71s with SC35 or DAP in nucleoskeleton preparations from bipolar and multipolar hippocampal neurons. Nucleoskeleton preparations of cultured hippocampal neurons at 21 DIV were used for immunostaining assays (see Materials and Methods section). Nucleoskeleton preparations of bipolar or multipolar hippocampal neurons were double stained with either Dp71d (Dys-2 Ab, green color, panel A) or Dp71f (5F3 Ab, green color, panel B); and SC35, α-DG (K8), β-DG (JAF), α1-, α2-DB (D124), β-DB (β-Brevin), and α-SYN (C4) Ab, respectively (red color, panel A and B). The colocalization between each Dp71 and SC35 or DAP was more evident in nuclear speckles of bipolar than multipolar hippocampal neurons, except for α1-, α2-DB (panel A and B). Yellow regions indicate the colocalization of Dp71s with SC35 or with each DAP in nuclear speckles. The colocalization rate of each Dp71 with SC35 or DAP is shown in the panel C. Values are means ± SEM, ٭٭p˂0.01. Scale bar = 5 μm.

Previously, it has been described that the nuclear speckles are tightly associated to the nucleoskeleton [34, 35], also known as nuclear scaffold or nuclear matrix [36, 37]. To further demonstrate if Dp71d or Dp71f nuclear colocalization with DAP detected in nuclear speckles, is associated to the nucleoskeleton, we obtained nucleoskeleton preparations from these neuronal cultures (Fig 6). Again, both Dp71s colocalized with SC35, α-DG, β-DG, α1-, α2-DB, β-DB and α-SYN, in nucleoskeleton preparations of bipolar and multipolar hippocampal neurons, respectively (Fig 6A and 6B). Furthermore, we measured the colocalization rate of Dp71 isoforms with DAP, and found that it was more evident in nucleoskeleton preparations, as compared to intact cells (Figs 6C and 5C, respectively). It is important to note, that Dp71d was better colocalized with β-DB and Dp71f with α1-DB, α2-DB in the nucleoskeleton preparations (Fig 6C); and these data are consistent with the IP assays (Fig 4).

These results demonstrated that the Dp71d-DAPC and Dp71f-DAPC were localized in nuclear speckles, and associated to nucleoskeleton, from bipolar GABAergic and multipolar Glutamatergic hippocampal neurons, respectively.

### Heat shock treatment effects the distribution of Dp71s in nuclear speckles

**Fig 7:**
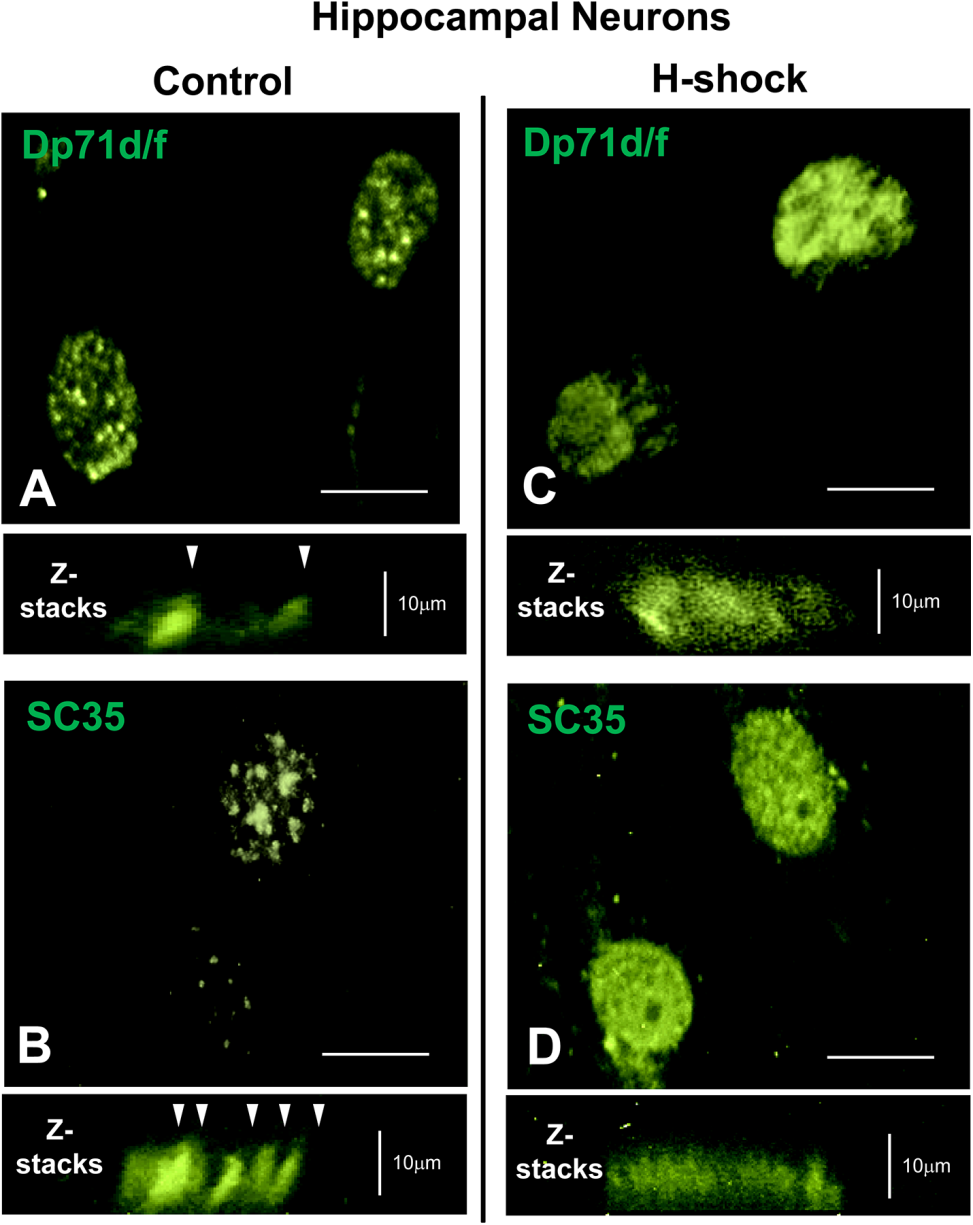
Localization of Dp71s in nuclear speckles of cultured hippocampal neurons. Cultured hippocampal neurons at 21 DIV were treated or non-treated with heat shock (see Materials and Methods section). Subsequently, were fixed and immunoreacted with Mandra-1 Ab (which detects both Dp71s isoforms, panel A and C) or anti-SC35 Ab that stains nuclear splicing speckles (panel B and D), respectively. In non-treated neurons (Control), Dp71d, Dp71f and SC35 were clearly localized in nuclear speckles structures or nuclear rod structures (white arrowheads, Z-stacks). In contrast, when neurons were heat shocked at 45°C for 15 min (H-shock), the localization of Dp71d, Dp71f and SC35 were dispersed, and speckles appear to be poorly stained or empty (panel C and D, respectively; Z-stacks). Scale bar = 10 μm.

To further corroborate whether Dp71s staining patters indeed are localized to nuclear speckles, we studied the pattern distribution of Dp71s under heat shock stress conditions, at 45°C for 15 min, to characterize if this condition, differentially alter the distribution of Dp71s as has been shown for SC35 protein [38]. The nuclear granules stained with anti-Dp71d/f (Mandra-1) or anti-SC35 Ab were observed as grains or pellets in control neurons (no heat shock treated, Fig 7A and 7B), while in heat shock treated neurons (H-shock), the granules were diffusely stained with both Ab (Fig 7C and 7D, respectively).

Taken together, these findings demonstrate that nuclear Dp71s are mainly localized in nuclear speckles from hippocampal neurons.

## Discussion

We have previously reported the intranuclear localization of Dp71d and Dp71f, in granular structures of cultured neurons from hippocampus. Both isoforms were colocalized with neurofilaments and with protein kinase C β_II_ (PKCβII) in nuclear speckles-like structures [28]. It has been described the presence of Dp71d-DAP complexes in the nuclei of non-neuronal cells [39–41], however, Dp71d-DAP and Dp71f-DAP complexes have not been detected in the nucleoskeleton of primary cultures of hippocampal neurons.

Herein, we demonstrated that Dp71d or Dp71f and DAP were expressed in the nucleus of primary hippocampal neurons cultured from 1h to 21 DIV, where Dp71d was more abundant in the nuclei of bipolar cells while Dp71f was more abundant in the nuclei of multipolar cells (Figs 1, 2 and 5). It is known that bipolar cells are mainly GABAergic neurons, and multipolar cells are predominantly Glutamatergic neurons, and has been suggested that nuclear Dp71s-DAPC may be linked to the regulation of the neurotransmitter function, at their respective synapses [17]. These groups of proteins integrate specific Dp71s-DAP complexes in nucleoskeleton from the cultured hippocampal neurons (Fig 4). Nuclear expression of both Dp71s varied along the temporal course of hippocampal neuronal culture; but, the highest level of nuclear expression of both Dp71s was at 10 DIV. After this time, the level of expression of nuclear Dp71d was maintained through the 15 DIV, but at 21 DIV, a decrease of this isoform was observed; while the level of nuclear expression of Dp71f drops at 15 and 21 DIV, to a similar level reached at 2 DIV (Fig 1C). Therefore, Dp71s may participate in the neuronal differentiation process, during the time course of the culture of hippocampal neuronal cells, as has been suggested for Dp71d in N1E-115 and PC12 cell lines [42, 43]. Here we report for the first time the nuclear localization of both Dp71s isoforms d and f in primary hippocampal neuronal cells, since it was only been described the nuclear localization of Dp71d in neuroblastome cell lines, but not Dp71f [42, 43]. Therefore we were able to characterize the nuclear localization pattern of both Dp71s in different neuronal populations: Dp71d was mainly localized to the nucleus of GABAergic neuronal cells, while Dp71f was mainly localized to the nucleus of Glutamatergic neurons (Fig 1).

Of note, is the fact that the protein complexes, Dp71d-DAPC and Dp71f-DAPC, colocalized in nuclear speckles of primary hippocampal cells (Figs 5 and 7). Moreover, the localization of Dp71s-DAPC, in nuclear speckles suggests, its participation in the regulation of alternative splicing and the co-activation of transcription [44], as it has been proposed for other nuclear proteins containing WW domains [45, 46].

Nuclear localization signal in Dp71 has been characterized in the ZZ domain in non-neuronal cells [47]. The specific nuclear localization of the each Dp71-DAP complex, observed here in GABAergic or Glutamatergic hippocampal neurons, appears to be related to specific synaptic functions of each type of neuron. It has been shown that highly mobile protein nuclear factors (HMG) are ubiquitous non-histone proteins that bind to DNA and regulate the expression of neuron-specific genes [48]. It was also demonstrated that β-DB interacts with some nuclear factors (iBRAF/HMG20a and BRAF35/HMG20b) [49]. iBRAF and BRAF35 have opposite roles: iBRAF activates REST-responsive genes through the modulation of histone methylation and BRAF35 mediates the repression of neuron-specific genes through remodeling chromatin structure [50, 51]. Accordingly, Dp71-DAP complexes may be regulating the co-activation or co-repression of neural specific genes [49], through the specific dystrobrevin isoform associated to each complex. In addition, β-DB binds to and represses the promoter of synapsin I, a marker for neuronal differentiation [49]. Synapsin I, is a neuronal phosphoprotein implicated in synaptogenesis and the modulation of neurotransmitter release [52, 53].

The main structural nuclear protein components are lamins A/C and actin, which are linked to some cytoskeleton proteins [54–56]. The findings reported here, indicate that Dp71s are bound to lamins A/C and actin (S3 Fig), therefore to the nucleoskeleton, thus Dp71s, may regulate the interactions cytoskeleton/nucleoskeleton [57]. It is possible that nuclear Dp71 isoforms may auto-modulate their function at the plasma and synaptic membranes [58–60]. As structural proteins, Dp71s may also participate in maintaining the nuclear structure and spatial compartmentalization required for specific nuclear functions. It has been shown that Dp71d is necessary to maintain the integrity of the nuclear architecture in C2C12 cell line [47]. Hence, nuclear alterations that affect their localization or the signaling of these protein complexes, may impact the function and viability of neurons.

Our data show that nNOS colocalized with Dp71d and Dp71f, showing a non-granular pattern, suggesting a different nuclear localization. nNOS is recruited to the nucleus, through its association with α-SYN, where it interacts with the Sp1 nuclear factor, and decreases the superoxide dismutase expression [61, 62]. Therefore, nNOS could participate in other signaling processes in the neuronal nucleus, such as the protection of neuronal cells, against oxidative stress [63].

β-DG is a key protein with versatile functions [64], that participates in cell signaling during cell polarity or cancer progression [65, 66]. Its signaling functions are associated to the presence of over 40 protein interactive domains, and 19 functional motifs, present at the β-DG C-terminal region rich in proline residues [67]. β-DG is an HMG protein and is associated to emerin, lamins A/C and B1, SC35, p80-coilin, and Nopp140 proteins in nucleus of HeLa cells and C2C12 cells, respectively [68, 69], suggesting that it may have a dynamic regulatory function. Thus, it is not surprising that we found that β-DG is associated to Dp71s-DAPC in the nucleoskeleton fraction by co-immunoprecipitation studies of primary cultures of hippocampal neuronal cells.

In prostate cancer, β-DG is tyrosine phosphorylated and translocated to the nucleus, where changes the gene transcriptional activity during the progression of cancer [70]. Consequently, β-DG is a multifunctional protein that participates in signaling from the plasma membrane to the cytoskeleton, that may also participates in the signaling processes from cytoskeleton/nucleoskeleton [71]. Thus, based on the data presented herein, we suggest that β-DG might participate in the transcription and regulation of genes involved in nuclear signaling processes and neuronal differentiation. The role of β-DG in the regulation of Dp71s complexes with DAP in neurons, remains to be elucidated.

**Fig 8:**
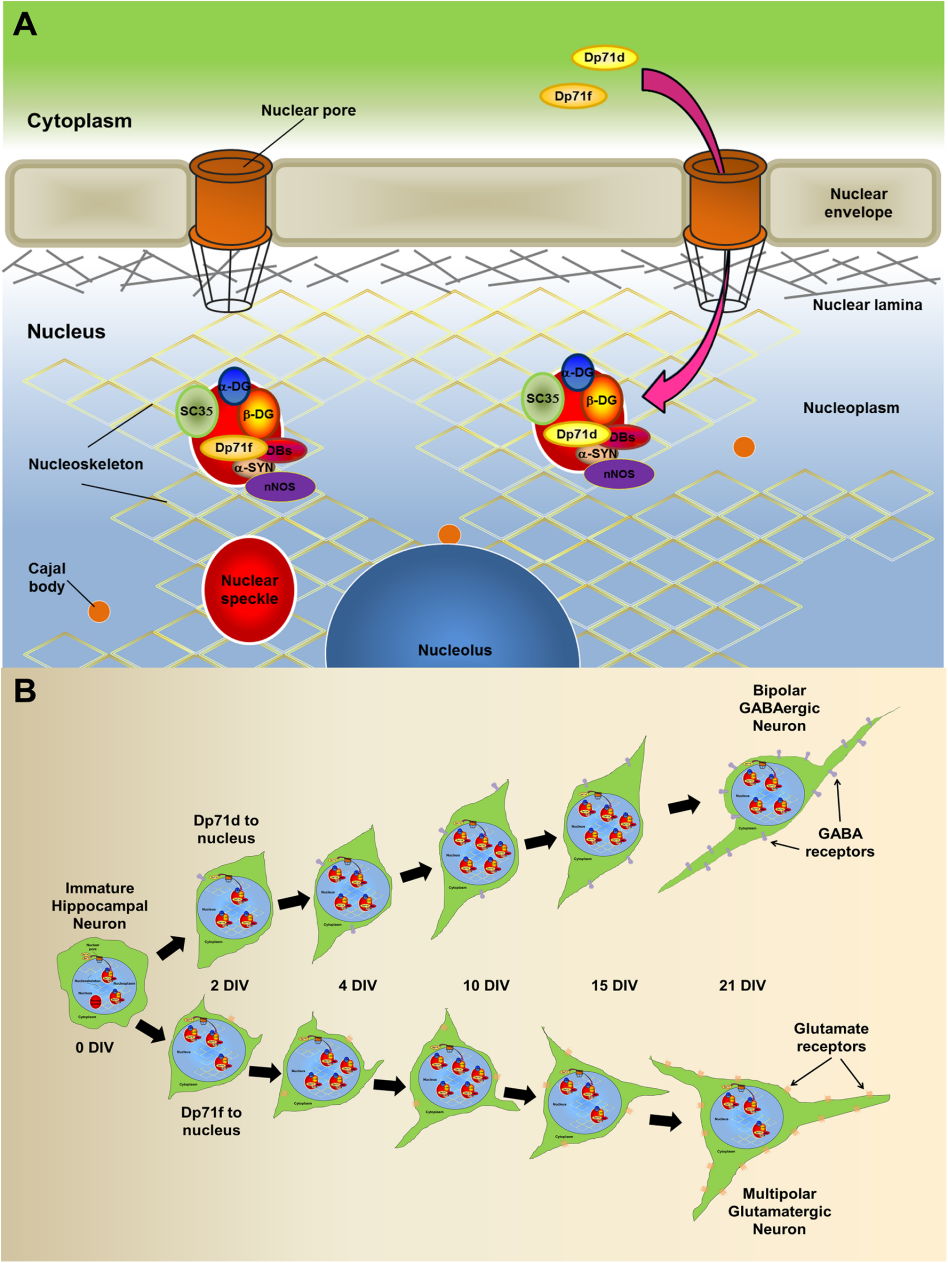
(A) Hypothetical model showing the nuclear localization of Dp71s-DAPC in hippocampal neurons. This model shows, the localization of Dp71d/f-DAP complexes in the nucleus of cultured hippocampal neurons at 21 DIV. Dp71d, Dp71f, and DAP (β-DG, α1-DB, β-DB, α2-DB, and α-SYN), as well as, nNOS, integrate protein complexes. Dp71d/f-DAP complexes were mainly localized in splicing speckle structures, which are anchored to the nucleoskeleton, possibly through structural proteins such as: lamins A/C and actin (see discussion for more details). (B) Representation of the neuronal differentiation and the nuclear expression pattern of the Dp71 isoforms d and f along the curse of the primary culture. Here we show the progression of neuronal differentiation throw the days of culture from 1h to 21 days, the nuclear localization of the Dp71 isoforms, and the expression phenotypic markers of GABAergic or Glutamatergic neurons. Thus Dp71d is primarily expressed in the nucleus of GABAergic neuronal cells, while Dp71f is preferentially expressed in the nucleus of Glutamatergic neurons.

Taken together our results, we propose a hypothetical model (Fig 8), in which, either Dp71 isoform could enter the nucleus throw the nuclear pore, and locate to the nuclear speckles, where it could form complexes with DAP, and interact with SC35, possibly modulating transcription and splicing (Fig 8A). Additionally we proposed that the differential nuclear localization of the Dp71 isoforms between the two principal neuronal phenotypes could be related to its role in neuronal differentiation (Fig 8B). In this model we show the progression of neuronal differentiation throw the days of culture from 1h to 21 days, the nuclear localization of the Dp71 isoforms, and the expression phenotypic markers of GABAergic or Glutamatergic neurons. Thus Dp71d is primarily expressed in the nucleus of GABAergic neuronal cells, while Dp71f is preferentially expressed in the nucleus of Glutamatergic neurons. Mouse lacking full length Dystrophin Dp427, or the short C-terminal isoforms Dp71s, show altered presynaptic ultrastructure in excitatory (Glutamatergic) hippocampal synapses, which may contribute to the intellectual disability characteristic of the X-linked DMD [59], and supports our observation about the possible contribution of Dp71s expression in the acquisition of the neurotransmitter phenotype (GABAergic vs Glutamatergic) during hippocampal neuronal differentiation.

In conclusion, in this study we demonstrate that: (i) Dp71d, Dp71f and DAP are localized in the nuclei of hippocampal neurons cultured at 21 DIV, (ii) Dp71d and Dp71f form complexes with DAP in neuronal nucleoskeleton, (iii) Dp71d-DAPC and Dp71f-DAPC are localized in speckles of nucleoskeleton from cultured hippocampal neurons (see hypothetical model in Fig 8), (iv) the complexes of Dp71d-DAPC and Dp71f-DAPC were detected in nuclear speckles of different neuronal phenotypes: GABAergic and Glutamatergic, respectively. The critical question about the functions of these sophisticated nuclear multi-protein complexes need to be understood in the total cellular context, therefore this work opens an interesting avenue concerning the function of these multi-protein complexes.

## Supporting Information

S1 ARRIVE Checklist ARRIVE chesklist. The ARRIVE (Animal Research: Reporting of In Vivo Experiments) guidelines for reporting animal studies.
(PDF)

S1 Fig Colocalization of Dp71s with DAPI in the nucleus of primary hippocampal neuronal cells. Primary hippocampal neuronal cells were immunolabeled with specific antibodies to Dp71s (see methods), and counterstained with DAPI. Colocalization was measured from confocal images using the LAS AF software from Leica.
(TIF)

S2 Fig Dp71s are the main DMD gene products in nuclear fraction from hippocampal neurons. Protein samples from cultured hippocampal neurons at 21 DIV: total extract (Total), and hippocampal nuclei (Nuclear) were analyzed by WB with Mandra-1 Ab (which detects both Dp71 isoforms). The Dp71d and Dp71f were the main dystrophin isoforms detected in the nuclear fraction, compared with the total extract (Dp427). Densitometric analysis is shown in panel B. The Dp71d expression was more robust than Dp71f in the nuclear fraction. Values are means ± SEM, ٭٭p˂0.01.
(TIF)

S3 Fig Coimmunoprecipitation of Dp71s with Lamins A/C and Actin in hippocampal neurons. Nucleoskeleton fractions were IP with Mandra-1 Ab (that detects both Dp71s), and shows that Lamins A/C (Lam A/C) and Actin interact with both Dp71 isoforms. As control for non-specific interactions, immunoprecipitation with CD4 (see Table 1), a non-related protein Ab was performed (IP-Unrelated Ab).
(TIF)
