# Supplementary figures and images for: Novel Nuclear Protein Complexes of Dystrophin 71 Isoforms in Rat Cultured Hippocampal GABAergic and Glutamatergic Neurons
Source: PLoS One. 2015 Sep 17;10(9):e0137328. doi: 10.1371/journal.pone.0137328 (PMC4574971; doi:10.1371/journal.pone.0137328)

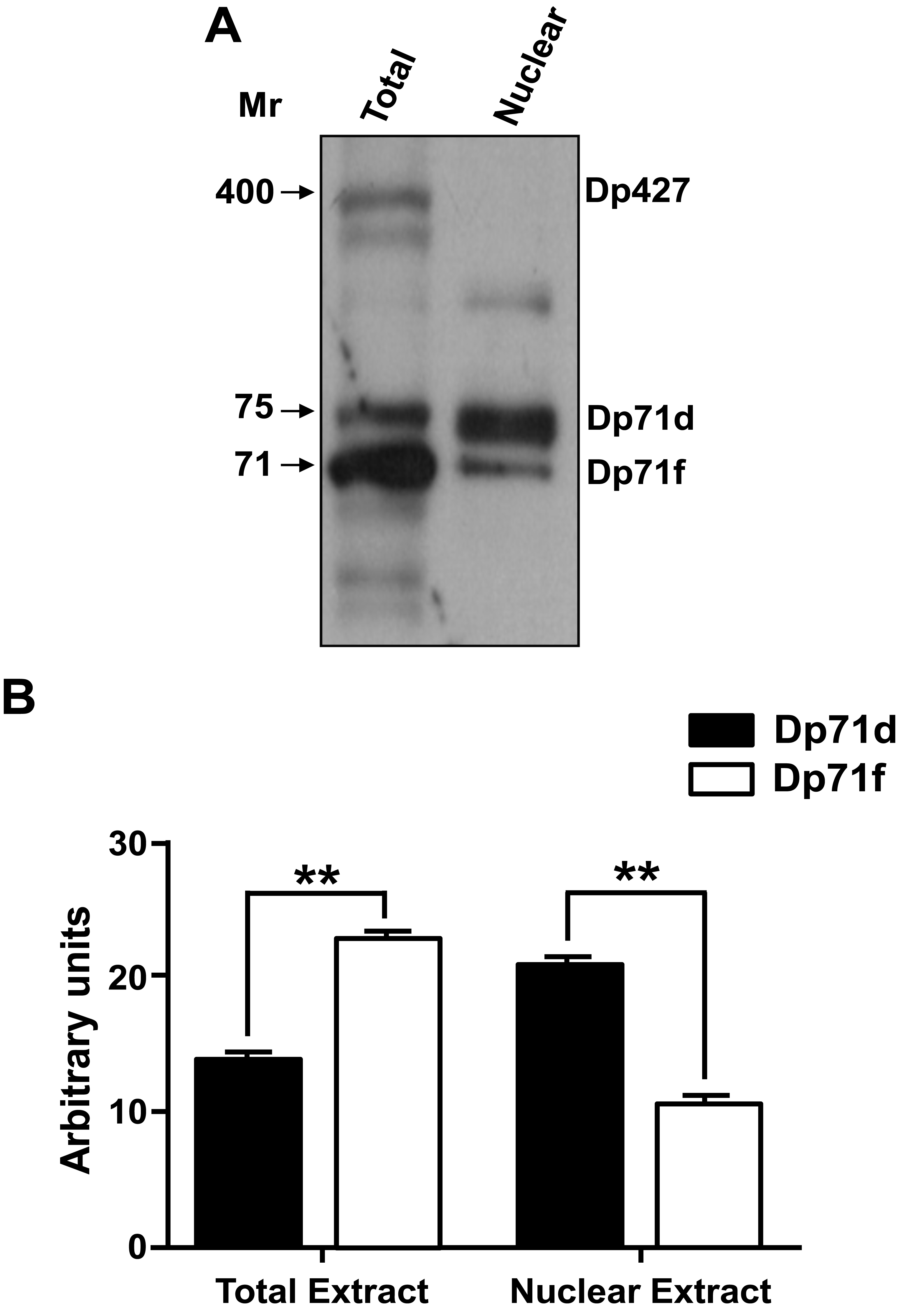

Supplement: S1 Fig — Primary hippocampal neuronal cells were immunolabeled with specific antibodies to Dp71s (see methods), and counterstained with DAPI. Colocalization was measured from confocal images using the LAS AF software from Leica. (TIF) [file pone.0137328.s002.tif]

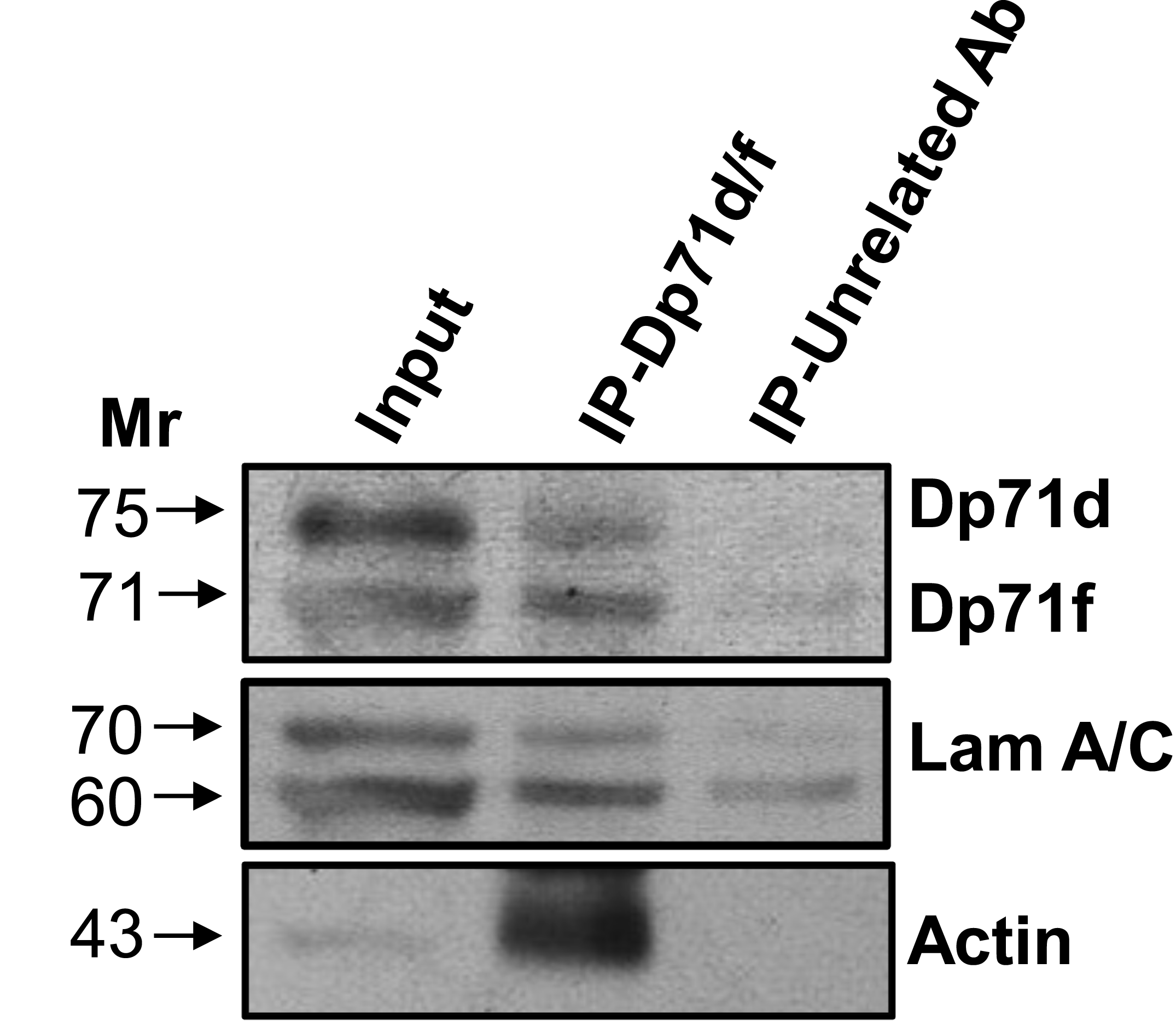

Supplement: S2 Fig — Protein samples from cultured hippocampal neurons at 21 DIV: total extract (Total), and hippocampal nuclei (Nuclear) were analyzed by WB with Mandra-1 Ab (which detects both Dp71 isoforms). The Dp71d and Dp71f were the main dystrophin isoforms detected in the nuclear fraction, compared with the total extract (Dp427). Densitometric analysis is shown in panel B. The Dp71d expression was more robust than Dp71f in the nuclear fraction. Values are means ± SEM, ٭٭p˂0.01. (TIF) [file pone.0137328.s003.tif]

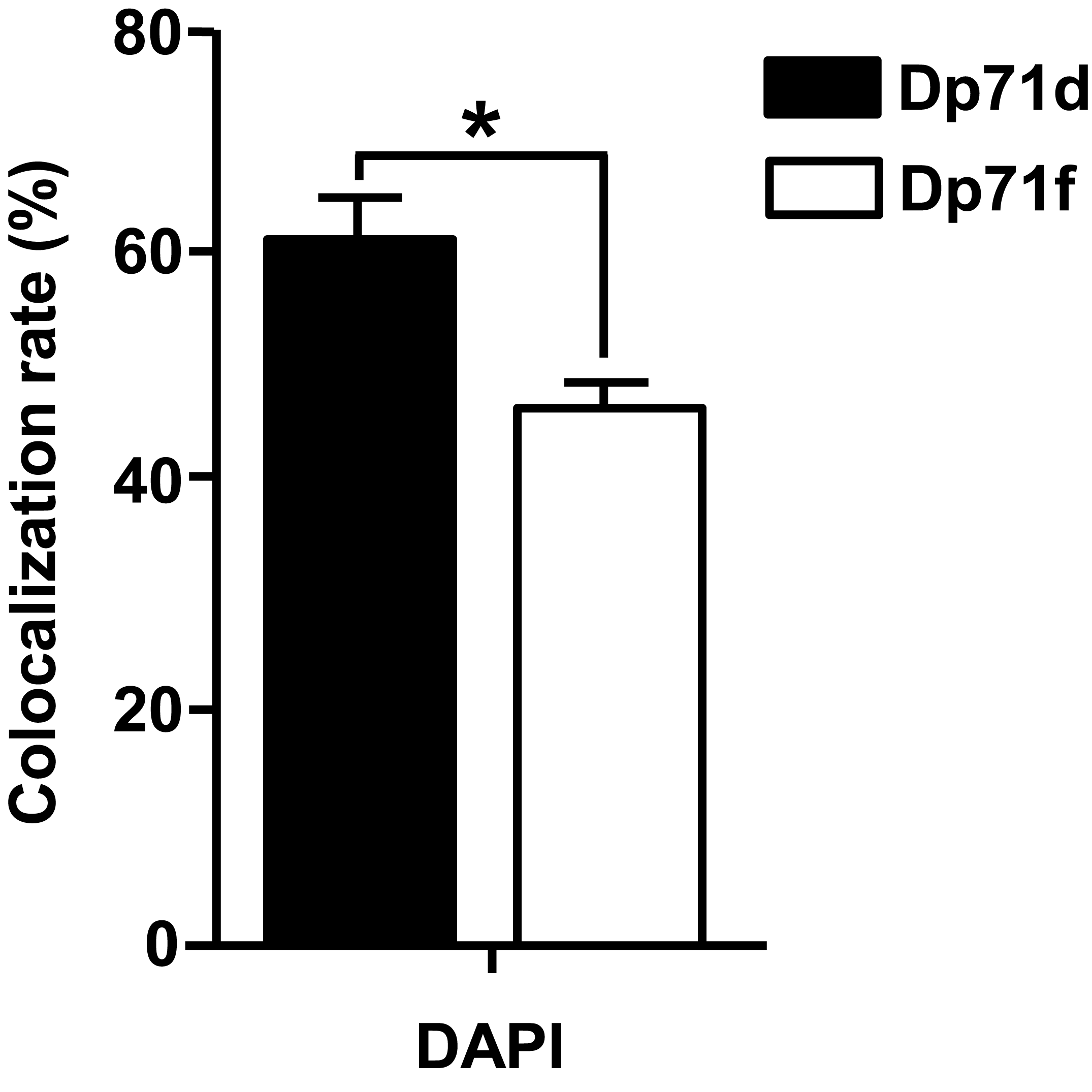

Supplement: S3 Fig — Nucleoskeleton fractions were IP with Mandra-1 Ab (that detects both Dp71s), and shows that Lamins A/C (Lam A/C) and Actin interact with both Dp71 isoforms. As control for non-specific interactions, immunoprecipitation with CD4 (see Table 1), a non-related protein Ab was performed (IP-Unrelated Ab). (TIF) [file pone.0137328.s004.tif]
